# Pharmacokinetic properties of the temozolomide perillyl alcohol conjugate (NEO212) in mice

**DOI:** 10.1093/noajnl/vdaa160

**Published:** 2020-11-20

**Authors:** Hee-Yeon Cho, Steve Swenson, Thu Zan Thein, Weijun Wang, Neloni R Wijeratne, Nagore I Marín-Ramos, Jonathan E Katz, Florence M Hofman, Axel H Schönthal, Thomas C Chen

**Affiliations:** 1 Department of Neurosurgery, Keck School of Medicine, University of Southern California, Los Angeles, California, USA; 2 Lawrence J. Ellison Institute for Transformative Medicine, University of Southern California, Los Angeles, California, USA; 3 Department of Molecular Microbiology and Immunology, Keck School of Medicine, University of Southern California, Los Angeles, California, USA; 4 Department of Pathology Keck School of Medicine, University of Southern California, Los Angeles, California, USA

**Keywords:** glioblastoma, HPLC, NEO212, pharmacokinetic, temozolomide

## Abstract

**Background:**

NEO212 is a novel small-molecule anticancer agent that was generated by covalent conjugation of the natural monoterpene perillyl alcohol (POH) to the alkylating agent temozolomide (TMZ). It is undergoing preclinical development as a therapeutic for brain-localized malignancies. The aim of this study was to characterize metabolism and pharmacokinetic (PK) properties of NEO212 in preclinical models.

**Methods:**

We used mass spectrometry (MS) and modified high-performance liquid chromatography to identify and quantitate NEO212 and its metabolites in cultured glioblastoma cells, in mouse plasma, brain, and excreta after oral gavage.

**Results:**

Our methods allowed identification and quantitation of NEO212, POH, TMZ, as well as primary metabolites 5-aminoimidazole-4-carboxamide (AIC) and perillic acid (PA). Intracellular concentrations of TMZ were greater after treatment of U251TR cells with NEO212 than after treatment with TMZ. The half-life of NEO212 in mouse plasma was 94 min. In mice harboring syngeneic GL261 brain tumors, the amount of NEO212 was greater in the tumor-bearing hemisphere than in the contralateral normal hemisphere. The brain:plasma ratio of NEO212 was greater than that of TMZ. Excretion of unaltered NEO212 was through feces, whereas its AIC metabolite was excreted via urine.

**Conclusions:**

NEO212 preferentially concentrates in brain tumor tissue over normal brain tissue, and compared to TMZ has a higher brain:plasma ratio, altogether revealing favorable features to encourage its further development as a brain-targeted therapeutic. Its breakdown into well-characterized, long-lived metabolites, in particular AIC and PA, will provide useful equivalents for PK studies during further drug development and clinical trials with NEO212.

**Key Points**

- We developed HPLC methods that allowed identification and quantitation of NEO212 and its metabolites in cells, plasma, and tissue.
- NEO212 preferentially concentrates in brain tumor tissue over normal brain tissue and has a higher brain:plasma ratio than TMZ.
- PK properties of NEO212 are favorable and support further development toward clinical testing.

**Importance of the Study**

NEO212 is the conjugate of temozolomide (TMZ) to perillyl alcohol (POH). Our previous studies had demonstrated its superior efficacy compared to TMZ in a variety of tumor cell types, including MGMT-positive and other TMZ-resistant variants. This current report is significant because it is the first time that the direct mechanism, potency, brain penetration, and metabolism of NEO212 have been directly measured and compared to TMZ. NEO212 stabilizes the prodrug TMZ, and allows better blood–brain barrier penetrance (up to 3×), greater cellular uptake and increased potency (up to 10×). Its mechanism of action remains based on DNA alkylation, with AIC as its main breakdown product, and myelodysplasia as its primary toxicity. Our goal is to get NEO212 to the clinic to be used in metastatic and primary brain cancers.

NEO212 is a novel small-molecule anticancer agent currently undergoing preclinical development. It was generated by covalent conjugation of perillyl alcohol (POH) to the alkylating agent temozolomide (TMZ). Oral TMZ is the current chemotherapeutic standard of care for the treatment of patients with newly diagnosed glioblastoma (GB), where it is administered concomitantly with radiotherapy and subsequently as monotherapy during the adjuvant phase.^1,2^ TMZ acts as a pro-drug; it undergoes spontaneous hydrolysis at physiological pH into its active metabolite, 5-(3-methyl triazen-1-yl) imidazole-4-carboxamide (MTIC).^3^ This product is then rapidly degraded into a methyldiazonium ion, representing the reactive species that methylates the DNA, and the inactive metabolite 5-aminoimidazole-4-carboxamide (AIC), which is stable in plasma for >24 h and is excreted through the kidneys.^3,4^ The interaction of the methyldiazonium ion with DNA yields a variety of alkylated bases, of which O^6^-methylguanine (O^6^MeG) is considered the most cytotoxic lesion.^5^

Several resistance mechanisms have been recognized that can protect cells from the cytotoxic impact of TMZ treatment. Foremost is overexpression of O^6^-methylguanine DNA methyltransferase (MGMT), a DNA repair enzyme that specifically removes the added methyl group from O6MeG, thereby eliminating the prime cytotoxic lesion set by TMZ.^5^ Separately, lack of an efficient DNA mismatch repair (MMR) system can provide TMZ resistance, because O6MeG lesions require functional MMR in order to trigger the double-strand breaks that lead to apoptosis.^6^ Other DNA lesions, in particular alkylation of N-purines by TMZ, are generally restored by base-excision repair (BER), and therefore increased activity of the BER system can provide an additional layer of resistance to TMZ. Based on this variety of molecular defenses against TMZ, it is not surprising that in clinical practice most GB patients experience recurrences, where the tumor has become unresponsive to further treatment with TMZ.^3,7^ Better treatments are urgently needed, and to address this medical need we have been developing NEO212, which might be able to overcome some of the shortcomings of TMZ.^8,9^

In NEO212, TMZ has been conjugated to POH, a naturally occurring monoterpene and metabolic product of limonene. It is commonly found in the essential oils of several plants and fruits, such as peppermint, spearmint, cherries, and celery seeds. POH has long been reported to have anticancer effects in vitro and in vivo.^10^ While initially it was characterized as an inhibitor of Ras oncoprotein, additional pleiotropic effects were described, including inhibition of telomerase activity and aggravation of endoplasmic reticulum stress,^11,12^ altogether exerting these effects primarily in cancer cells where these components already are overly active. The cancer therapeutic activity of POH was further investigated in several phase I/II clinical trials, where it was administered orally in rather large quantities.^10^ However, the documented anticancer activity was unimpressive, and patients found the continuous daily regimen hard to tolerate. As a result, oral POH did not enter clinical practice. As an alternative, an intranasal formulation of POH was developed, and currently ongoing clinical trials are revealing well-tolerated, promising activity of this approach in patients with recurrent GB.^13,14^ The 2 main metabolites of POH in mammals were identified as perillic acid (PA) and dihydroperillic acid (DHPA)^15^ while the parental molecule, POH itself, was difficult to detect, presumably due to its very short biological half-life.^15–17^

In conjugating POH with TMZ, we desired to create a molecule with beneficial features contributed by both of its components, that is, alkylation function and pleiotropic impairment of cancer cell-specific hallmarks. In addition, our in silico analysis of NEO212 predicted effective penetration of the blood–brain barrier (BBB) and higher brain-targeted activity as compared to TMZ.^18^ Thus, results from computer modeling made NEO212 particularly attractive for development as a brain cancer agent.

NEO212 has revealed robust cancer therapeutic activity in a wide variety of in vitro and in vivo tumor models. In mouse tumor models, NEO212 displayed intracranial activity not only against GB,^9,19^ but also against brain-metastatic breast cancer xenografts.^18^ Beside intracranial tumors, NEO212 was also effective against peripheral cancers, such as subcutaneous melanoma,^20^ nasopharyngeal carcinoma,^21,22^ ovarian carcinoma,^23^ lung cancer,^24–26^ and cutaneous T-cell lymphoma.^27^ In addition, NEO212 exerted activity even against TMZ-resistant cells, irrespective of the underlying resistance mechanism. At the same time, treatment was well tolerated by the tumor-bearing animals and no signs of toxicity could be detected. Intriguingly, whenever NEO212 was compared side-by-side to treatments with TMZ or POH individually, the conjugated compound consistently displayed significantly greater anticancer activity. Combination treatments with mixtures of TMZ with POH were unable to mimic the superior effects of NEO212, demonstrating that the potency of the conjugated compound is greater than the sum of its parts.^8,9,19,20^

Although NEO212 has been extensively studied at the preclinical level, no plasma or brain PK data are available. As well, its in silico-predicted efficient BBB penetration has not been confirmed experimentally, nor has it been established whether treatment with NEO212 would indeed generate the predicted metabolites in vivo. This latter point is of particular relevance, because knowledge of metabolites and the availability of respective assays to measure them are needed in preparation for clinical studies of NEO212. In the following, we are presenting the analytical identification and PK measurement of NEO212 and its key metabolites in plasma and brain of mice.

## Materials and Methods

### Cell Lines

The human glioma cell lines U251, LN229, and T98G were purchased from the American Tissue Culture Collection (ATCC). The U251-derived TMZ-resistant (TR) cell line was developed in this laboratory^9^ and the mouse glioma cell line GL261 was provided by Dr. Linda M. Liau (University of California Los Angeles). Chemoresistant glioma cancer stem cells USC02 were isolated from a patient with glioblastoma as described previously.^19^ Cell culture conditions were as described elsewhere^28^ and further details are provided in Supplementary Materials.

### Pharmacological Agents

NEO212 was synthesized by Norac Pharma under cGMP conditions as a crystalline powder and was kindly provided by NeOnc Technologies, Inc. A stock solution (1M in DMSO) was further diluted with a 50:50 mixture (vol:vol) of glycerol and ethanol before being administered to animals by oral gavage. For cell culture treatments, the stock solution was diluted with cell culture medium. TMZ was purchased from Sigma Aldrich and dissolved in DMSO to a concentration of 50 mM before administered to animals or cell culture. DMSO, glycerol, and ethanol were purchased from Sigma Aldrich.

### Xenografts

All animal protocols were approved by the Institutional Animal Care and Use Committee of USC, and all rules and regulations were followed during experimentation. Intracranial implantation of mouse glioma cells (GL261) was performed as previously described.^28^ At 12 days postimplantation, animals were randomly separated into different treatment groups with 3 animals in each group. The drugs were administered by oral gavage for a one-time treatment. At different time points thereafter, animals were euthanized. Blood was collected immediately, followed by perfusion and harvest of brains. Brains were then separated into their right and left hemispheres, with and without tumor tissue, respectively. All the samples were processed for high-performance liquid chromatography (HPLC) analysis.

### NEO212 and AIC Detection in Urine and Feces

Using a metabolic cage, the excrements from 3 mice were collected over 3 separate time periods, from 0–1 h, 0–4 h, and 0–24 h. Solid material was homogenized in PBS and filtered through a 0.22-µm filter with the resultant filtrate again filtered through a 4k molecular weight cutoff filter. Hundred microliters of filtrate was then placed in 200 µl acetonitrile for NEO212 analysis and 100 µl of filtrate was mixed with 200 µl 5% acetonitrile in water for AIC analysis. Urine was treated in a similar fashion without homogenization, but using the same filter units. The volume of the samples was standardized with water.

### Half-Life Study of NEO212 and TMZ in Mice

Female 6–8-week-old C57BL/6 mice, weighing approximately 30 g, were obtained from Charles River Laboratories. The mice were grouped as follows: (1) no treatment, (2) vehicle treated, (3) NEO212 treated (50 mg/kg), and (4) TMZ treated (50 mg/kg). The drugs were administered by oral gavage for a one-time treatment, and blood samples were collected at different time points thereafter: 15, 60, 120, and 240 min (*n* = 3). The half-life was determined by a nonlinear fit of the concentrations over time using one-phase decay.

### Analytical Procedures for NEO212 and Its Metabolites

See Supplementary Materials for detailed methods of mass spectrometry (MS) and HPLC.

### Statistical Analysis

The calculation of plasma pharmacokinetic parameters was carried out using a non-linear regression analysis from the point of maximum observed concentration. The concentrations for each sample were calculated from the area under the curve (AUC) values for the AIC peaks detected. AUC values were calculated using a standard curve of NEO212 and AIC, which was obtained by spiking samples of tissue, substances, or fluids with these agents. The *P*-values were determined by a 2-way ANOVA analysis of the data and *P* < .05 was considered significant.

## Results

### The Chemical Structure and Breakdown Products of NEO212

**Figure 1.**
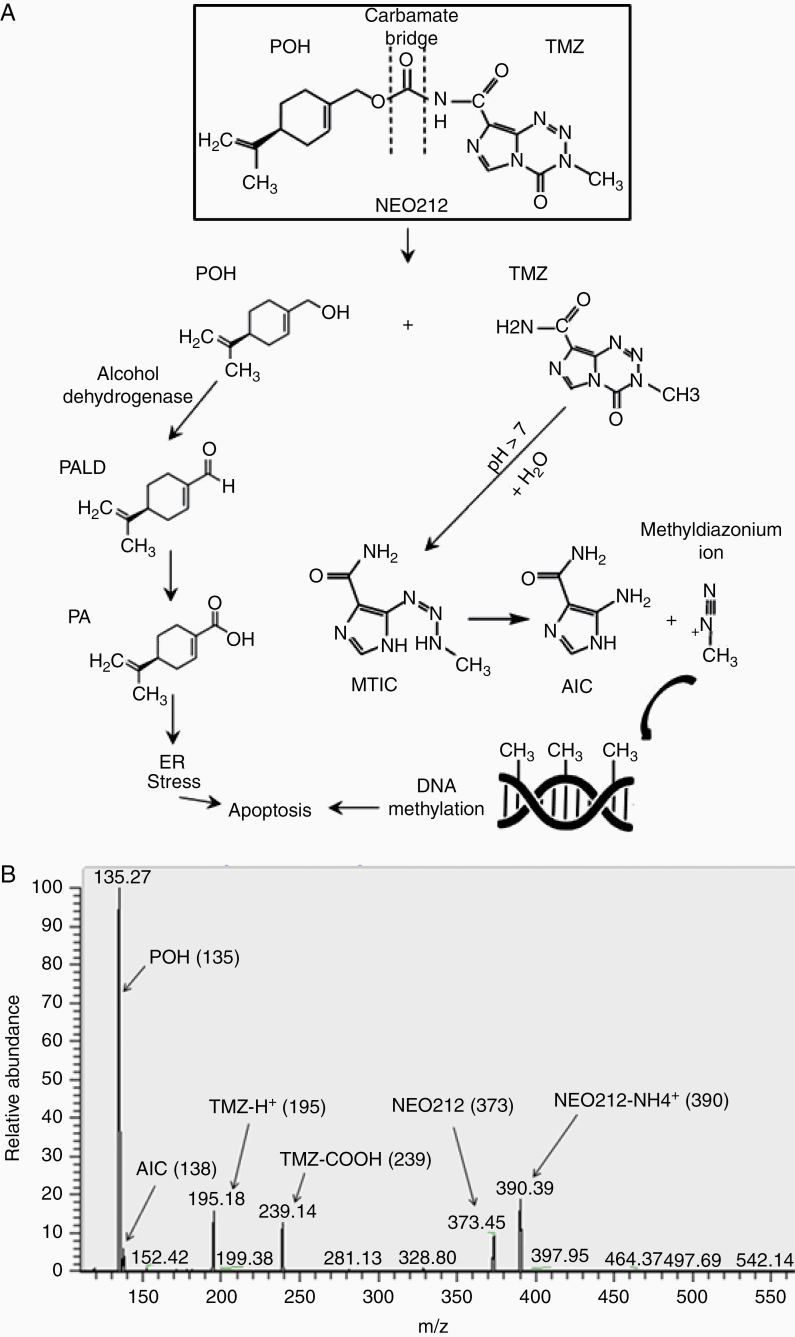
The chemical structure and predicted breakdown products of NEO212. (A) NEO212 is a conjugation product of POH and TMZ, where the 2 components are connected via a carbamate bridge. NEO212 might decay into POH and TMZ. The methyldiazonium ion alkylates O6MeG DNA lesions and triggers apoptosis. POH and PA contribute to tumor cell death via aggravation of ER stress. (B) NEO212 breakdown product analysis using MS.

NEO212 was synthesized by covalently conjugating TMZ to POH via carbamate bonding. The resultant product was authenticated by ^1^H and 13C nuclear magnetic resonance (NMR) spectroscopy and by comparison to specified reference standards, and verified to be 99.6% pure. NEO212’s CAS number is 1361198-79-9, with 372.379 molecular weight and a molecular formula of C_17_H_20_N_6_O_4_. It is an off-white to light yellow to light brown crystalline solid that readily dissolves in organic solvents. Based on the known metabolites of TMZ and POH, which have been extensively documented, we predicted that NEO212 would break down in a biological system as outlined in Figure 1A. The carbamate bridge between TMZ and POH would be amenable to hydrolysis by esterases, presumably yielding both of the individual partner molecules, TMZ and POH, and possibly slightly modified versions thereof, depending on the exact cleavage site. TMZ is known to convert to short-lived MTIC, which is followed by degradation into long-lived AIC plus the DNA alkylating methyldiazonium ion.^3,4^ POH is known to be metabolized to short-lived perillyl aldehyde (PALD), followed by conversion to more stable PA.^29,30^

We employed a modified MS approach to begin our investigation of NEO212 breakdown. Using this method, we were able to identify NEO212 (Figure 1B). In addition, we could also document the presence of TMZ and POH, confirming that both individual partner molecules could be derived from the conjugated parent molecule. The mass spectrogram also showed a peak at 138 (identified as AIC), a peak at 390 (identified as NEO212-NH_4_^+^, a reaction product with the solvent), and a peak at 239 (TMZ-carboxylic acid). To achieve faster and routine quantification of these molecules, especially in view of our preclinical and future clinical pharmacokinetic demands, we developed modified HPLC-based analytical approaches. As shown in Supplementary Figure S1, these procedures were able to readily detect and quantitate NEO212, TMZ, POH, and importantly AIC and PA, which are stable metabolites and therefore suitable as indirect readouts of overall drug exposure in a biological system. In all, both MS and HPLC demonstrated that NEO212 could yield its 2 main partner molecules and their metabolites, enabling us to apply these validated methods to biological systems, that is, cultured cells and mouse-derived tissues.

### Cellular Uptake of NEO212

**Figure 2.**
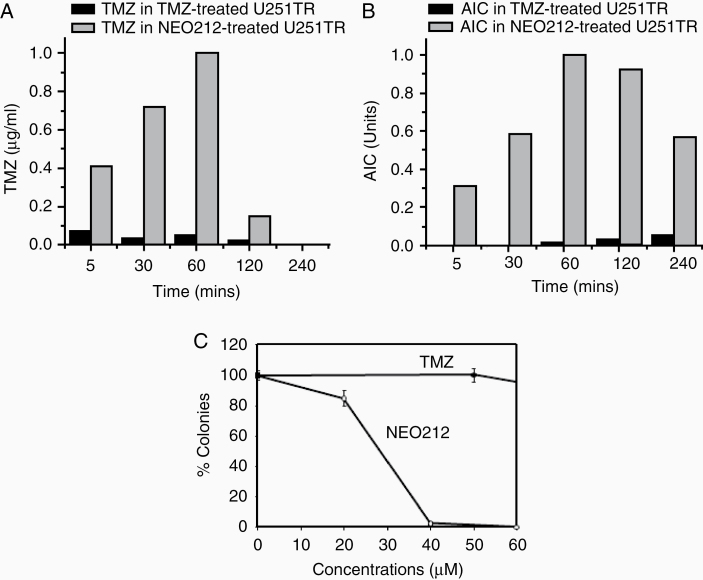
Cellular uptake of NEO212 compared to TMZ. U251TR cells were treated with 100 μM of either NEO212 or TMZ. (A) The amount of intracellular TMZ was measured using MS. (B) The amount of intracellular AIC was measured using MS. All differences were statistically significant. (C) Colony forming assay was performed using different concentrations of drugs using U251TR cells.

An intriguing observation in all our prior studies was the consistently documented ability of NEO212 to demonstrate high anticancer potency than TMZ, both in vitro and in vivo. To investigate our hypothesis that differential drug uptake might be involved, we treated human U251TR glioblastoma cells with NEO212 or the same concentration of TMZ, followed by analysis of intracellular concentrations of TMZ and AIC over time. As shown in Figure 2A and B, the amounts of TMZ and AIC inside cells were substantially greater after treatment with NEO212 (>10-fold higher) than after treatment with TMZ. All differences were statistically significant. The intracellular presence of TMZ could be verified up to 120 min after start of drug treatment, but became undetectable at 240 min (Figure 2A). In comparison, the intracellular amount of AIC remained high and easily detectable up to 240 min (Figure 2B), consistent with this metabolite’s known greater stability as compared to TMZ. Nonetheless at all time points, exposure of cells to NEO212 resulted in much more TMZ and AIC inside cells than exposure to equal concentrations of TMZ, indicating that uptake of NEO212 is more efficient than uptake of TMZ. As AIC may represent an indirect readout for production of the active DNA alkylating species, that is, the diazonium ion, higher levels of AIC in NEO212-treated cells over TMZ-treated cells provide a reasonable rationale to explain NEO212’s greater anticancer impact. We compared the cytotoxic difference between NEO212 and TMZ in U251TR cells, which clearly correlates with their intracellular presence, that is, higher intracellular levels of NEO212 (and metabolites) correlate with higher cytotoxic impact in these tumor cells (Figure 2C). We also used different glioma cell lines (LN229 and T98G) to monitor TMZ and AIC level after TMZ or NEO212 treatments. As shown in Supplementary Figure S2A and B, exposure of cells to NEO212 consistently resulted in much more TMZ and AIC inside cells than exposure to equal concentrations of TMZ, indicating that uptake of NEO212 was more efficient than uptake of TMZ in all the cell lines we tested. Additionally, we used chemoresistant glioma cancer stem cells (GSCs) to compare cellular uptake of NEO212 and TMZ in relation to these drug’s cytotoxic impact. Consistent with the above findings with established cell lines, cellular uptake of NEO212 was substantially greater than uptake of TMZ in GSCs; correspondingly, while these cells were resistant to TMZ, NEO212 was able to exert pronounced cytotoxic impact (Supplementary Figure S3).

### Half-Life of NEO212 in Mouse Plasma

**Figure 3.**
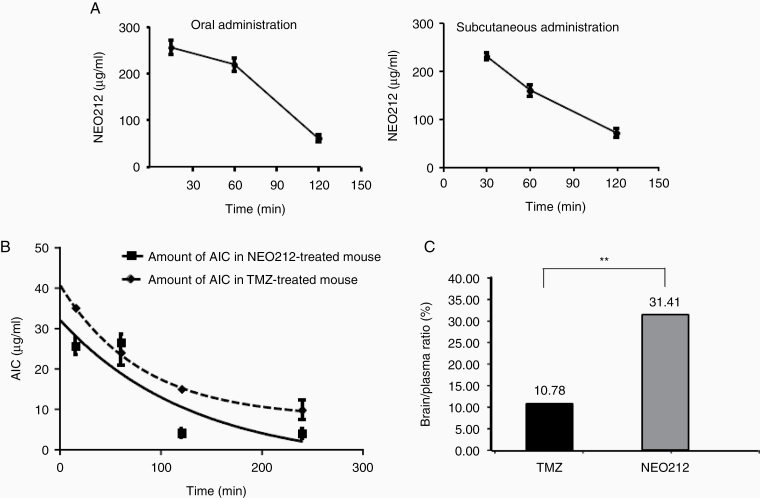
Half-life of NEO212 or AIC in mouse plasma. (A) NEO212 concentrations were measured in plasma by HPLC after oral administration (left) or subcutaneous administration (right) of NEO212 (50 mg/kg) to C57BL/6 mice. (B) AIC concentrations in plasma were measured using HPLC after administration of NEO212 (50 mg/kg) or TMZ (50 mg/kg) to C57BL/6 mice. The concentrations for each sample were converted from the AUC for the AIC peak detected. (C) Brain:plasma ratio of NEO212 and TMZ. Two asterisks (**): *P* < .015.

We next determined pharmacokinetic properties of NEO212 in mice. To measure the half-life of NEO212 in plasma, a single dose of 50 mg/kg NEO212 was administered by oral gavage, followed by blood collections at different time points thereafter. Plasma measurements revealed peak NEO212 concentrations at 15 min after dosing, which represented the earliest measured time point (Figure 3A, left). Subsequently, NEO212 concentrations decreased over the following 2 h and became undetectable at 24 h (24-h time point not shown). The calculated half-life was 94 min. NEO212 plasma concentrations after subcutaneous administration of NEO212 (50 mg/kg) was also measured (Figure 3A, right). The results demonstrated that NEO212 reached its peak concentration in the plasma in 30 min and decreased with time and no NEO212 was detected after 24 h. The results were similar under both conditions, indicating comparable NEO212 half-life. We next studied the plasma kinetics of AIC, after administration of NEO212 and TMZ. Mice received a single dose of 50 mg/kg NEO212 or TMZ by oral gavage, followed by blood collections at different time points thereafter. Figure 3B shows that peak AIC concentrations were reached within 15 min of NEO212 dosing, followed by a slow decrease over time and significant amounts of AIC still detectable after 240 min. The calculated half-life of AIC was 90 min. AIC levels after dosing of mice with TMZ followed a similar pattern, although at slightly lower overall levels and shorter half-life of 80 min. In all, these measurements revealed somewhat greater in vivo stability of NEO212 and higher levels of AIC, as compared to TMZ, and therefore provided additional aspects that might underlie NEO212’s superior anticancer potency.

### Neuropharmacokinetics of NEO212

Our prior in silico analysis predicted that NEO212 would be able to cross the BBB more effectively than TMZ.^18^ Studies with tumor-bearing mice showed that NEO212 indeed was more active than TMZ against brain-localized disease,^8^ further supporting the initial prediction. To quantitate this effect, we measured brain:plasma ratios of both agents after treatment of mice with either NEO212 or TMZ. As shown in Figure 3C, the brain:plasma ratio of NEO212 was 3 times greater than that of TMZ, which was in agreement with our earlier studies and validated yet another desirable attribute of NEO212, especially in view of applications in glioblastoma therapy.

**Figure 4.**
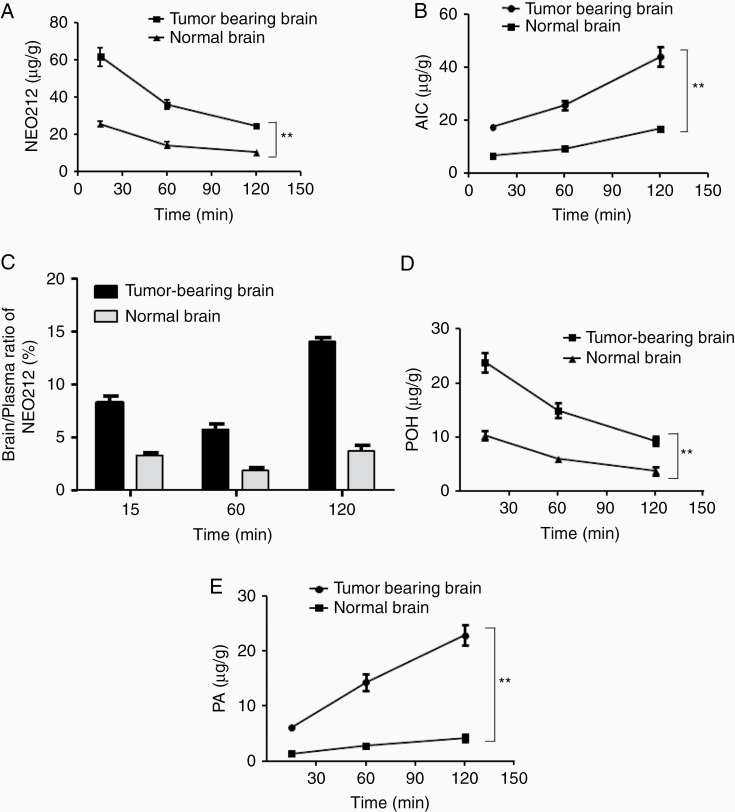
Differential uptake of NEO212 or AIC in normal and tumor-bearing brains. (A) NEO212 concentrations in the tumor-bearing brain and normal brain (**P < .015). (B) AIC concentrations in the tumor-bearing brain and normal brain (**P < .001). (C) Overall comparison of brain to plasma ratios of NEO212 (*P* < .015). (D) POH concentrations in the tumor-bearing brain and normal brain (**P < .015). (E) PA concentrations in the tumor-bearing brain and normal brain (**P < .001).

We next investigated whether the uptake of NEO212 would be different in a normal brain as compared to one harboring a brain tumor. C57BL/6 mice, with or without intracranially implanted syngeneic GL261 mouse glioma cells, received a single dose or oral NEO212. Their brains were collected at different times thereafter and analyzed for NEO212 and AIC. The results show that the concentrations of both molecules were consistently about 2 to 3 times higher in tumor-bearing brains than in normal brains. The highest levels of NEO212 were detected at 15 min after drug treatment, with a subsequent slow decline over the following 2 h (Figure 4A). In comparison, AIC levels displayed the inverse kinetic, that is, the metabolite continuously increased during the 2-h period of measurement (Figure 4B). The significantly greater presence of these molecules in tumor-bearing brains, in particular 3 times more AIC (the indirect indicator of DNA alkylating discharge), would be considered a benefit for future therapeutic purposes.

We also investigated whether the above observed differential brain tumor uptake of NEO212 would be reflected in differential brain:plasma ratios at the same time points. For this purpose, we compared NEO212 concentrations from tumor-bearing brains and normal brains to those measured in the plasma obtained from the same animals. As shown in Figure 4C, the brain: plasma ratio was consistently 2–3-fold higher in brains with tumors, further confirming that NEO212 preferentially enters brain tumors over normal brain tissue.

We also measured POH and PA concentrations in the same samples described above. Figure 4D shows that the POH concentrations were about 2–3 times higher in tumor-bearing brains than in normal brains. The highest POH concentration was reached in 15 min. The POH metabolite, PA levels however showed inverse kinetics like AIC and PA continuously increased and reached its highest concentrations after 2 h (Figure 4E). The significantly greater presence of these molecules in tumor-bearing brains, in particular 3 times more AIC (the indirect indicator of DNA alkylating discharge), bodes well for future therapeutic purposes.

### Excretion of NEO212

**Figure 5.**
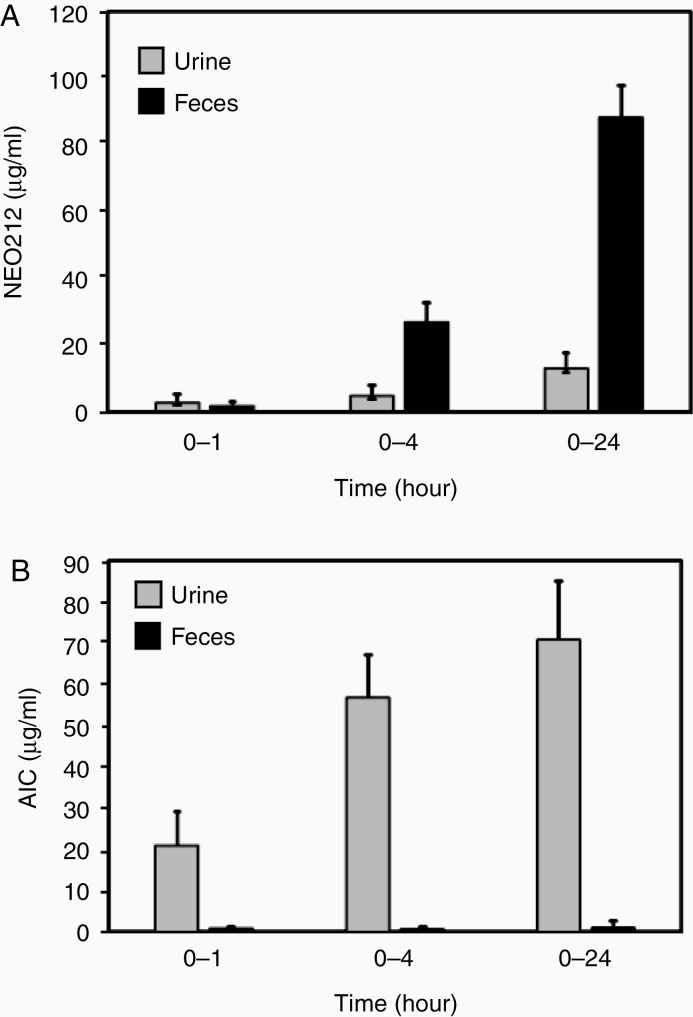
Excretion of NEO212 with oral administration. (A) Time profile of NEO212 in urine and feces. (B) Time profile of AIC in urine and feces.

The excretion kinetics of NEO212 and AIC were determined by evaluating feces and urine collected during specific time periods after oral delivery of a single dose of NEO212 to mice. Figure 5A shows that NEO212 was excreted preferentially in feces rather than urine, with the majority of it being excreted after the first 4 h postdosing. Figure 5B shows that AIC was present in the urine and barely detectable in feces, with the majority of it being excreted within the first 4 h postdosing.

## Discussion

NEO212 is a hybrid molecule that was generated by conjugation of POH to TMZ. In all studies, NEO212 revealed stronger anticancer effects than its individual components, TMZ or POH by themselves, or the sum of its parts.^8,9,20,21^ To further characterize the NEO212 molecule and understand its superior anticancer function, we now performed analytical measurements of its metabolism in vitro and in vivo.

Both TMZ and POH have undergone extensive clinical characterization in the past. Therefore, metabolic pathways for these 2 agents are very well established.^3,15^ For NEO212, we hypothesized that esterase activity, which is abundantly present in biological systems, would hydrolyze the carbamate bond between the conjugated molecules to release POH and TMZ, which would then be subjected to the established metabolic processes. Based on results presented in the current report, this model indeed appears to be correct.

Using MS, we were able to identify NEO212, as well as its predicted key breakdown products TMZ and POH, in an aqueous solution (Figure 1B). AIC was identified also, which indirectly pointed to the generation of a methyldiazonium ion, which is the reactive species known to methylate DNA. MTIC, the intermediate between TMZ and AIC, was not detected, presumably due to its particularly short-lived nature. POH was readily identified by MS, although none of its metabolites. This was unsurprising, because POH requires specific enzymatic activities for stepwise oxidation to its metabolites PALD and PA, whereas in comparison the decay of TMZ to MTIC and AIC is a nonenzymatic process that spontaneously takes place in aqueous solution and neutral pH. It is noteworthy that the events of MS fragmentation take place in the presence of elevated energy molecular ions in a high vacuum, and therefore are not entirely reflective of solution chemistry of biological samples. For instance, breakage of the carbamide bond under MS conditions reflects its bombardment with electrons, as esterases were not present in the reaction conditions. This is also indicated by our detection of TMZ-caboxylic acid (TMZ-COOH) as a reaction species. Despite this limitation, MS analysis provided a preliminary portrait of NEO212 chemistry, which was further illuminated and complemented by HPLC-based analytical methods.

We employed published HPLC protocols^16,31–33^ and successfully modified them toward the specific detection of NEO212, TMZ, POH, AIC, and PA from biological environments (Supplementary Figure S1), such as cell lysates, mouse plasma, and brain tissue after exposure to NEO212. In cell culture experiments, we established that the intracellular concentrations of TMZ and AIC were significantly higher in NEO212-treated cells than in TMZ-treated cells (Figure 2). Thus, despite identical drug concentrations in the culture medium, cellular uptake of NEO212 appears much more efficient than uptake of TMZ. Although we did not investigate details of the uptake mechanism, we deem it unlikely that this process involves prior extracellular decay of NEO212 into TMZ + POH, followed by POH-facilitated uptake of TMZ. This rationale is based on the fact that mere combination treatment of cells with individual components, that is, TMZ mixed with POH, is unable to mimic the substantially greater cytotoxic potency of NEO212. Thus, the presence of POH in the extracellular space is not able to increase the cytotoxic potency of TMZ over that of TMZ alone, excluding a model based on efficient co-transport of 2 separate molecules into cells. Rather, in view of POH’s amphipathic physiochemical character and its ability to intercalate into cellular membranes,^34^ we propose that NEO212 efficiently associates with the membrane bilayer, where the conjugated TMZ moiety is towed along and eventually separated from the compound molecule by intracellular esterases. While validation of this conjecture remains to be established, the unequivocally greater presence of intracellular AIC after NEO212 treatment is reflective of increased delivery of DNA-alkylating, apoptosis-inducing methyldiazonium ions, thereby providing a straightforward explanation as to NEO212’s greater cytotoxic potency over TMZ in cell culture.

The treatment of all brain-localized diseases is limited by poor BBB penetration of most therapeutics. Even TMZ shows only partial ability to penetrate the BBB, with a brain-to-plasma ratio in the range of 10%–25%, depending on the model used.^35,36^ In our previous studies, we had used a computer program that predicted in silico whether a given compound would enter the BBB and exert activity in the brain.^18^ By comparing NEO212 to TMZ, the program predicted superior performance by NEO212, which was further supported by results from in vivo studies, where we documented greater therapeutic impact of NEO212 as compared to TMZ in mouse brain tumor models.^8,9^ The results shown in Figure 3C now provide analytic quantitation of this effect by revealing that brain:plasma ratios of NEO212 are 3-times higher than those of TMZ, after oral gavage of mice. This result points to favorable characteristics of NEO212 if used for brain malignancy. A recognized side effect of TMZ therapy is myelosuppression of the bone marrow, which limits dosing of this oral drug. Based on NEO212’s alkylating properties, qualitative similar toxicities should be expected. However, based on the nonproliferative nature of brain cells, neurotoxicity should be low, and brain parenchyma should be able to tolerate increased drug dosages (as is the case with TMZ). Therefore, based on a 3 times increased brain:plasma ratio, one would envision the possibility of delivering greater alkylating impact to the brain, while at the same time keeping systemic exposure (in particular bone marrow) at an equivalent level to TMZ. Therefore, at similar systemic toxicity as TMZ, NEO212 would be expected to achieve greater therapeutic impact on brain cancers. So far, the preclinical experience in mouse models is in agreement with this prediction, as these studies have documented superior activity of NEO212 in brain cancer, along with undetectable systemic toxicities. Furthermore, preferential uptake of NEO212 by brain tumor tissue over normal brain tissue, and respectively increased levels of AIC in brain tumors (as demonstrated in Figure 4), indicate additional features that bode well for future clinical applications. Moreover, its activity in MGMT-positive GB^37^ cannot be overemphasized, as it may also be used to treat these patients who currently do not have much benefit from TMZ.

Our study further establishes plasma half-life of NEO212 (94 min, Figure 3), which is only slightly longer than what has been reported for TMZ.^38^ In view of repeat once-daily dosing, this fairly short half-life would not be expected to result in significant drug accumulation over time. Although this aspect will require further confirmation, the available data therefore suggest that a similar oral dosing schedule to TMZ would be appropriate in the clinical setting.

In summary, our study introduces HPLC-based methods for quantification of NEO212 and several of its metabolites. We investigated in vivo metabolism of this novel conjugate and characterized the presence of TMZ, POH, AIC, and PA. The latter 2 are of particular interest, because they are longer-lived, easy to detect, and derived from the same initial molecule at stochiometric ratios. As such, both AIC and PA represent suitable readouts for overall drug exposure. Their detection and quantification will support further studies on NEO212, and will become particularly useful during clinical trials, where drug exposure of patients requires careful characterization and monitoring.

## Supplementary Material

vdaa160_suppl_Supplementary_Materials

## Notes

**Authorship Statement.** H.-Y.C. designed and performed experiments, contributed to data collection and analysis, and drafted the manuscript. S.S. performed experiments and drafted the manuscript. T.Z.T. performed experiments and reviewed the manuscript. W.W. performed experiments. N.R.W. performed experiments. N.I.M.-R. contributed to study design, data analysis, and reviewed the manuscript. J.E.K. performed data analysis and interpretation of results. A.H.S. contributed to study design and data analysis, and finalized the manuscript. F.M.H. contributed to study design and data analysis. T.C.C. conceived of the study, acquired funding, contributed to study design, data analysis and supervision, and reviewed the manuscript. All authors approved the final version of the manuscript.

## References

[1] Stupp R, Hegi ME, Mason WP, et al.; European Organisation for Research and Treatment of Cancer Brain Tumour and Radiation Oncology Groups; National Cancer Institute of Canada Clinical Trials Group Effects of radiotherapy with concomitant and adjuvant temozolomide versus radiotherapy alone on survival in glioblastoma in a randomised phase III study: 5-year analysis of the EORTC-NCIC trial. Lancet Oncol. 2009;10(5):459–466. 19269895 10.1016/S1470-2045(09)70025-7

[2] Stupp R, Mason WP, van den Bent MJ, et al.; European Organisation for Research and Treatment of Cancer Brain Tumor and Radiotherapy Groups; National Cancer Institute of Canada Clinical Trials Group Radiotherapy plus concomitant and adjuvant temozolomide for glioblastoma. N Engl J Med. 2005;352(10):987–996. 15758009 10.1056/NEJMoa043330

[3] Newlands ES, Stevens MF, Wedge SR, Wheelhouse RT, Brock C Temozolomide: a review of its discovery, chemical properties, pre-clinical development and clinical trials. Cancer Treat Rev. 1997;23(1):35–61. 9189180 10.1016/s0305-7372(97)90019-0

[4] Tsang LL, Farmer PB, Gescher A, Slack JA Characterisation of urinary metabolites of temozolomide in humans and mice and evaluation of their cytotoxicity. Cancer Chemother Pharmacol. 1990;26(6):429–436. 2225314 10.1007/BF02994094

[5] Fahrer J, Kaina B O6-methylguanine-DNA methyltransferase in the defense against N-nitroso compounds and colorectal cancer. Carcinogenesis. 2013;34(11):2435–2442. 23929436 10.1093/carcin/bgt275

[6] Fu D, Calvo JA, Samson LD Balancing repair and tolerance of DNA damage caused by alkylating agents. Nat Rev Cancer. 2012;12(2):104–120. 22237395 10.1038/nrc3185 PMC3586545

[7] Weller M, Cloughesy T, Perry JR, Wick W Standards of care for treatment of recurrent glioblastoma–are we there yet? Neuro Oncol. 2013;15(1):4–27. 23136223 10.1093/neuonc/nos273 PMC3534423

[8] Chen TC, Cho HY, Wang W, et al. A novel temozolomide-perillyl alcohol conjugate exhibits superior activity against breast cancer cells in vitro and intracranial triple-negative tumor growth in vivo. Mol Cancer Ther. 2014;13(5):1181–1193. 24623736 10.1158/1535-7163.MCT-13-0882

[9] Cho HY, Wang W, Jhaveri N, et al. NEO212, temozolomide conjugated to perillyl alcohol, is a novel drug for effective treatment of a broad range of temozolomide-resistant gliomas. Mol Cancer Ther. 2014;13(8):2004–2017. 24994771 10.1158/1535-7163.MCT-13-0964

[10] Chen TC, Fonseca CO, Schönthal AH Preclinical development and clinical use of perillyl alcohol for chemoprevention and cancer therapy. Am J Cancer Res. 2015;5(5):1580–1593. 26175929 PMC4497427

[11] Cho HY, Wang W, Jhaveri N, et al. Perillyl alcohol for the treatment of temozolomide-resistant gliomas. Mol Cancer Ther. 2012;11(11):2462–2472. 22933703 10.1158/1535-7163.MCT-12-0321

[12] Sundin T, Peffley DM, Gauthier D, Hentosh P The isoprenoid perillyl alcohol inhibits telomerase activity in prostate cancer cells. Biochimie. 2012;94(12):2639–2648. 22902867 10.1016/j.biochi.2012.07.028

[13] da Fonseca CO, Schwartsmann G, Fischer J, et al Preliminary results from a phase I/II study of perillyl alcohol intranasal administration in adults with recurrent malignant gliomas. Surg Neurol. 2008;70(3):259–266; discussion 266-257. 18295834 10.1016/j.surneu.2007.07.040

[14] da Fonseca CO, Simão M, Lins IR, Caetano RO, Futuro D, Quirico-Santos T Efficacy of monoterpene perillyl alcohol upon survival rate of patients with recurrent glioblastoma. J Cancer Res Clin Oncol. 2011;137(2):287–293. 20401670 10.1007/s00432-010-0873-0 PMC11828245

[15] Phillips LR, Malspeis L, Supko JG Pharmacokinetics of active drug metabolites after oral administration of perillyl alcohol, an investigational antineoplastic agent, to the dog. Drug Metab Dispos. 1995;23(7):676–680. 7587953

[16] Hua HY, Zhao YX, Liu L, Ye QX, Ge SW High-performance liquid chromatographic and pharmacokinetic analyses of an intravenous submicron emulsion of perillyl alcohol in rats. J Pharm Biomed Anal. 2008;48(4):1201–1205. 18849133 10.1016/j.jpba.2008.08.015

[17] Ripple GH, Gould MN, Stewart JA, et al. Phase I clinical trial of perillyl alcohol administered daily. Clin Cancer Res. 1998;4(5):1159–1164. 9607573

[18] Chen TC, Da Fonseca CO, Schonthal AH Perillyl alcohol and its drug-conjugated derivatives as potential novel methods of treating brain metastases. Int J Mol Sci. 2016;17(9):1463. 10.3390/ijms17091463 PMC5037741 27598140

[19] Jhaveri N, Agasse F, Armstrong D, et al. A novel drug conjugate, NEO212, targeting proneural and mesenchymal subtypes of patient-derived glioma cancer stem cells. Cancer Lett. 2016;371(2):240–250. 26683773 10.1016/j.canlet.2015.11.040

[20] Chen TC, Cho HY, Wang W, et al. A novel temozolomide analog, NEO212, with enhanced activity against MGMT-positive melanoma in vitro and in vivo. Cancer Lett. 2015;358(2):144–151. 25524552 10.1016/j.canlet.2014.12.021

[21] Chen TC, Cho HY, Wang W, et al. Chemotherapeutic effect of a novel temozolomide analog on nasopharyngeal carcinoma in vitro and in vivo. J Biomed Sci. 2015;22:71. 26282951 10.1186/s12929-015-0175-6 PMC4539921

[22] Xie L, Song X, Guo W, et al. Therapeutic effect of TMZ-POH on human nasopharyngeal carcinoma depends on reactive oxygen species accumulation. Oncotarget. 2016;7(2):1651–1662. 26625208 10.18632/oncotarget.6410 PMC4811487

[23] Song X, Liu L, Chang M, et al. NEO212 induces mitochondrial apoptosis and impairs autophagy flux in ovarian cancer. J Exp Clin Cancer Res. 2019;38(1):239. 31174569 10.1186/s13046-019-1249-1 PMC6554966

[24] Chang M, Song X, Geng X, et al. Temozolomide-Perillyl alcohol conjugate impairs Mitophagy flux by inducing lysosomal dysfunction in non-small cell lung Cancer cells and sensitizes them to irradiation. J Exp Clin Cancer Res. 2018;37(1):250. 30326943 10.1186/s13046-018-0905-1 PMC6191917

[25] Song X, Xie L, Chang M, et al. Temozolomide-perillyl alcohol conjugate downregulates O6-methylguanin DNA methltransferase via inducing ubiquitination-dependent proteolysis in non-small cell lung cancer. Cell Death Dis. 2018;9(2):202. 29426908 10.1038/s41419-017-0193-2 PMC5833843

[26] Song X, Xie L, Wang X, et al. Temozolomide-perillyl alcohol conjugate induced reactive oxygen species accumulation contributes to its cytotoxicity against non-small cell lung cancer. Sci Rep. 2016;6:22762. 26949038 10.1038/srep22762 PMC4780103

[27] Silva-Hirschberg C, Hartman H, Stack S, et al. Cytotoxic impact of a perillyl alcohol-temozolomide conjugate, NEO212, on cutaneous T-cell lymphoma in vitro. Ther Adv Med Oncol. 2019;11:1758835919891567. 31839810 10.1177/1758835919891567 PMC6900611

[28] Cho HY, Thein TZ, Wang W, et al. The Rolipram-perillyl alcohol conjugate (NEO214) is a mediator of cell death through the death receptor pathway. Mol Cancer Ther. 2019;18(3):517–530. 30647121 10.1158/1535-7163.MCT-18-0465

[29] Elegbede JA, Flores R, Wang RC Perillyl alcohol and perillaldehyde induced cell cycle arrest and cell death in BroTo and A549 cells cultured in vitro. Life Sci. 2003;73(22):2831–2840. 14511768 10.1016/s0024-3205(03)00701-x

[30] Yeruva L, Pierre KJ, Elegbede A, Wang RC, Carper SW Perillyl alcohol and perillic acid induced cell cycle arrest and apoptosis in non small cell lung cancer cells. Cancer Lett. 2007;257(2):216–226. 17888568 10.1016/j.canlet.2007.07.020

[31] de Lima DC, Rodrigues SV, Boaventura GT, et al. Simultaneous measurement of perillyl alcohol and its metabolite perillic acid in plasma and lung after inhalational administration in Wistar rats. Drug Test Anal. 2020;12(2):268–279. 31800149 10.1002/dta.2722

[32] Kim H, Likhari P, Parker D, et al. High-performance liquid chromatographic analysis and stability of anti-tumor agent temozolomide in human plasma. J Pharm Biomed Anal. 2001;24(3):461–468. 11199225 10.1016/s0731-7085(00)00466-0

[33] Shen F, Decosterd LA, Gander M, Leyvraz S, Biollax J, Lejeune F Determination of temozolomide in human plasma and urine by high-performance liquid chromatography after solid-phase extraction. J Chromatogr B Biomed Appl. 1995;667(2):291–300. 7663702 10.1016/0378-4347(95)00040-p

[34] da Fonseca CO, Khandelia H, Salazar MD, Schönthal AH, Meireles OC, Quirico-Santos T Perillyl alcohol: dynamic interactions with the lipid bilayer and implications for long-term inhalational chemotherapy for gliomas. Surg Neurol Int. 2016;7:1. 26862440 10.4103/2152-7806.173301 PMC4722523

[35] Ostermann S, Csajka C, Buclin T, et al. Plasma and cerebrospinal fluid population pharmacokinetics of temozolomide in malignant glioma patients. Clin Cancer Res. 2004;10(11):3728–3736. 15173079 10.1158/1078-0432.CCR-03-0807

[36] Portnow J, Badie B, Chen M, Liu A, Blanchard S, Synold TW The neuropharmacokinetics of temozolomide in patients with resectable brain tumors: potential implications for the current approach to chemoradiation. Clin Cancer Res. 2009;15(22):7092–7098. 19861433 10.1158/1078-0432.CCR-09-1349 PMC2908372

[37] Marín-Ramos NI, Thein TZ, Cho HY, et al. NEO212 inhibits migration and invasion of glioma stem cells. Mol Cancer Ther. 2018;17(3):625–637. 29440289 10.1158/1535-7163.MCT-17-0591 PMC5935548

[38] Reyderman L, Statkevich P, Thonoor CM, Patrick J, Batra VK, Wirth M Disposition and pharmacokinetics of temozolomide in rat. Xenobiotica. 2004;34(5):487–500. 15370964 10.1080/00498250410001685737

