# Supplementary material for: Pharmacokinetic properties of the temozolomide perillyl alcohol conjugate (NEO212) in mice
Source: Neurooncol Adv. 2020 Nov 20;2(1):vdaa160. doi: 10.1093/noajnl/vdaa160 (PMC7764505; doi:10.1093/noajnl/vdaa160)
Supplement: vdaa160_suppl_Supplementary_Materials [file vdaa160_suppl_supplementary_materials.docx]

**Pharmacokinetic Properties of the Temozolomide Perillyl Alcohol Conjugate (NEO212) in Mice**

Hee-Yeon Cho, Steve Swenson, Thu Zan Thein, Weijun Wang,

Neloni R. Wijeratne, Nagore I. Marín-Ramos, Jonathan E. Katz, Florence M. Hofman, Axel H. Schönthal, Thomas C. Chen

**Supplementary Materials**

- **Supplementary Methods**
- **Supplementary Figure S1**
- **Supplementary Figure S2**
- **Supplementary Figure S3**

**SUPPLEMENTARY METHODS**

**Cell culture**

The human glioma cell lines LN229, T98G, U251, and U251TR, as well as the mouse glioma cell line GL261, were propagated in Dulbecco’s Modified Eagle Medium (DMEM) supplemented with 10% fetal bovine serum (FBS), 100 U/mL penicillin, and 0.1 mg/mL streptomycin in a humidified incubator at 37°C and a 5% CO_2_ atmosphere. USC02 glioma cancer stem cells were cultured in cancer stem cell culture (CSC) medium containing DMEM-F12 medium (Life Technologies, Grand Island, NY) with 1% penicillin-streptomycin, 1% B-27 (Life Technologies), 20 ng/ml EGF and FGF-2 (Peprotech, Rocky Hill, NJ).

**Colony Forming Assay (CFA)**

Glioma cells were seeded in 6-well plates at 200 cells/ well and allowed to adhere overnight. Subsequently, cells were treated with drugs for 48 hours; the medium was then removed and fresh medium (without drug) was added. Cells were incubated for an additional 7-10 days. At the termination of the assay, colonies were visualized by staining with 1% methylene blue in methanol for 4 hours. Dyes were washed out with water and then air dried. The stained colonies were counted. Percent colonies were calculated relative to untreated control cells. All experiments were performed in triplicate. Vehicle-treated cells were also included, but showed no difference to untreated cells.

**MTT cytotoxicity assay**

Glioma stem cells (GSCs) were seeded in 96 well plates in CSC medium containing BSA (Sigma Aldrich) equivalent to 10% FCS. After 24 hours, TMZ or NEO212 was added to the cells at different concentrations, and incubated for 72 hours. The MTT assay was performed according to the manufacturer’s protocol (Sigma Aldrich, St. Louis, MO). Absorbance was measured using a microtiter plate reader (Molecular Devices, Sunnyvale, CA) at 490 nm. Percent viability was calculated relative to untreated control cells. All experiments were performed in triplicate.

**Bioassay of NEO212 and its metabolites by mass spectrometry (MS)**

Cell lysates were stabilized with 100 mM ammonium acetate (AmAc) (pH 4.0), followed by a simple protein precipitation method with methanol to clean up the samples prior to analysis. After vortexing for 15 sec, the solutions were chilled at –20˚C for 20 min and centrifuged at 4˚C using 20817 xg for 20 min. The supernatant was directly injected into the MS instrument for analysis. Calibration curves for each drug were generated for quantification. Individual stock solutions of TMZ (400 μg/ml) (Sigma Aldrich), NEO212 (400 μg/ml) and angiotensin II (40 µg/ml; internal standard) were serially diluted with acidic methanol (methanol:1M AmAc = 90:10).

**HPLC Analysis**

All plasma or brain lysate samples (100 µl) were mixed with 200 µl acetonitrile and filtered with a 0.22 μm nylon filter (Nalgene) or a 4k MW cut-off filter. A 10-µl aliquot of filtrate was injected into an i-Series Plus Integrated HPLC System (Shimadzu, Columbia, MD) with an integrated photo-diode array detector (PDA). LabSolutions V5.87 SP1 software (Shimadzu) was used for data acquisition and instrument control. The isocratic separation of NEO212, TMZ, POH and PA was performed using a Roc C18 column (10 x 4.6 mm x 3 μm) (Restek Corporation, Bellefonte, PA) with a column temperature of 30ºC for 30 minutes. The mobile phase consisted of acetonitrile plus 0.1% trifluoroacetic acid (TFA): water plus 0.1% TFA (pH 4.0) (40:60 v/v). The AIC gradient separation utilized A (water-acetonitrile-trichloroacetic acid, 95:5:0.1, v/v/v) and B (water-acetonitrile-trichloroacetic acid, 5:95:0.1, v/v/v) 100% A to 100% B. Flow rate was 1.0 mL/min with a temperature of 30ºC. As internal standard, ibuprofen (Cayman Chemical, Ann Arbor, MI) was utilized.

**Plasma sample preparation**

Mice were euthanized and 300 µl of blood was collected and acidified by addition of 300 μl of citrate buffer (0.8% sodium citrate in H_2_O). The tube was gently inverted 8-10 times until thoroughly mixed. The samples were placed on ice and centrifuged at 4°C within 30 minutes. All samples were prepared in triplicate.

**Brain tissue homogenate preparation**

Mouse brain was washed once with ice-cold PBS and homogenized in 1 ml of ammonium acetate buffer (pH 4.0) in a tissue homogenizer. The lysate was centrifuged at 12,000 rpm for 15 minutes. The supernatant was collected and stored at –80˚C until analyzed by HPLC. All samples were prepared in triplicate.

**SUPPLEMENTARY FIGURES**

**
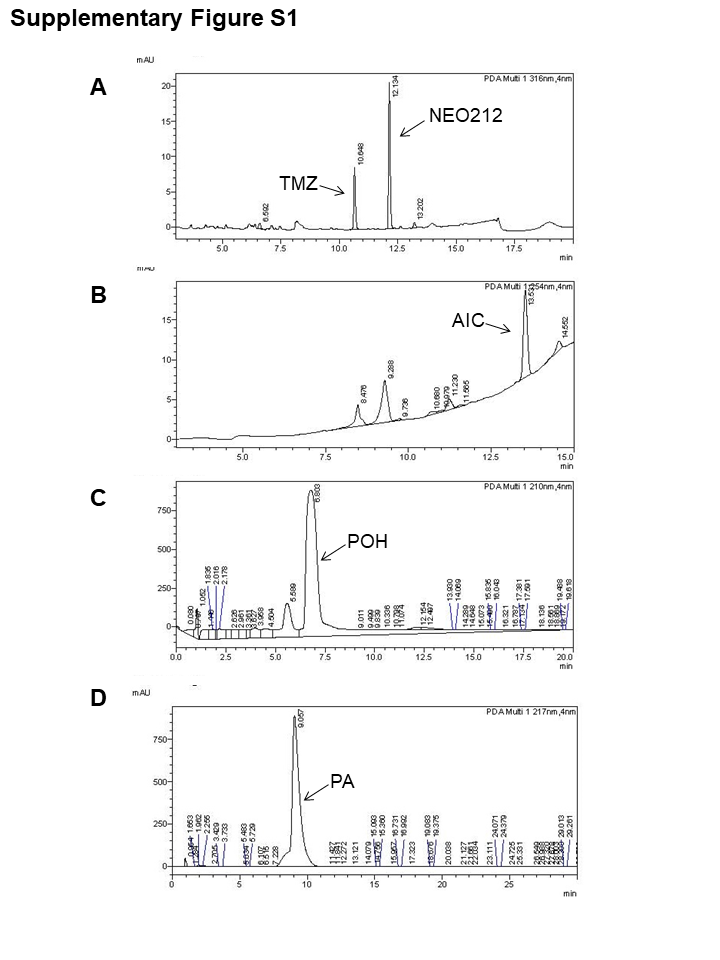
**

**Supplementary Figure S1.** **HPLC chromatograms of NEO212 and its breakdown products**

(A) NEO212 and TMZ was detected at 316 nm. (B) AIC was detected at 254 nm. (C) POH was detected at 210 nm. (D) PA was detected at 217 nm.

**
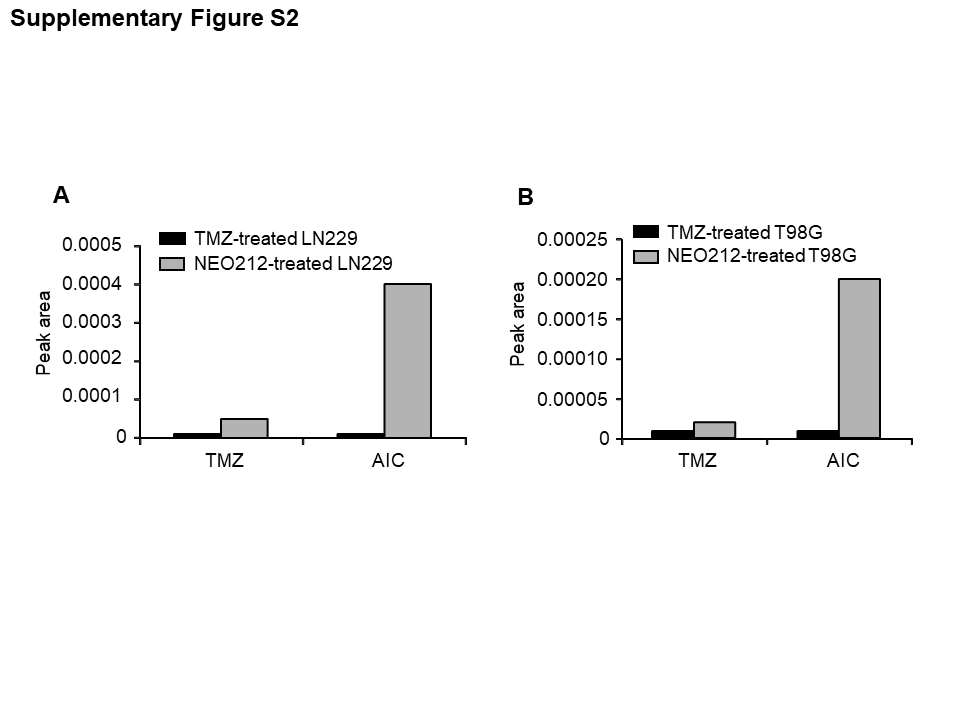
**

**Supplementary Figure S2. Cellular uptake of NEO212 compared to TMZ in LN229 and T98G glioma cell lines**

(A) The amount of intracellular TMZ or AIC in LN229 cells at 60 minutes after treatment with 100 μM of either NEO212 or TMZ was measured by mas spectroscopy (MS). (B) The amount of intracellular TMZ or AIC in T98G cells at 60 minutes after treatment with 100 μM of either NEO212 or TMZ was measured by MS. All differences between TMZ-treated cells and NEO212-treated cells were statistically significant (*p*<0.05).

**
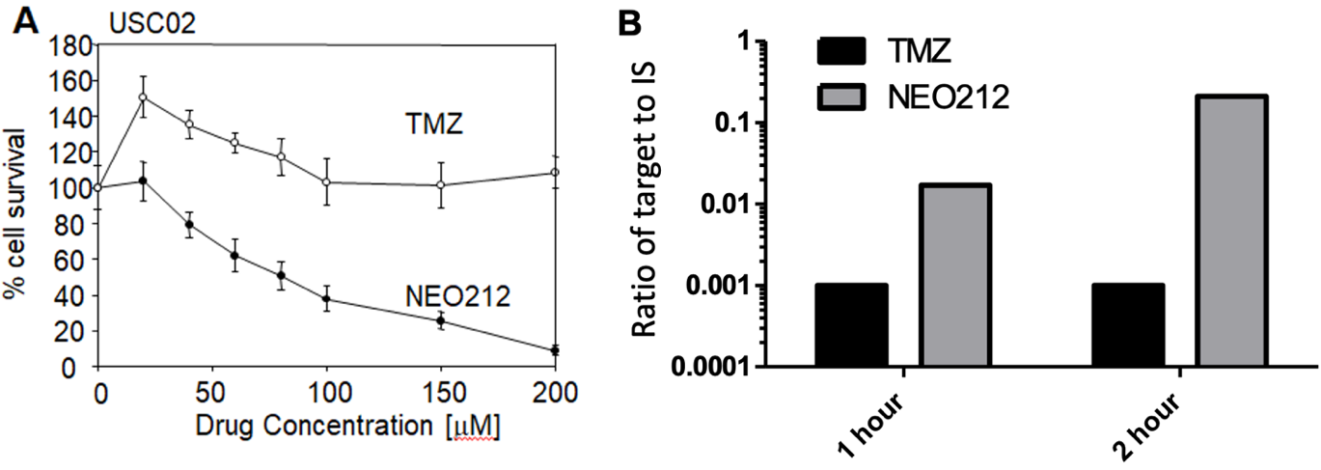
**

**Supplementary Figure S3. Cytotoxicity and cellular uptake of TMZ and NEO212 in chemoresistant glioma cancer stem cells USC02**

USC02 is highly drug-resistant glioma stem cell line isolated from a patient with glioblastoma. (A) MTT cytotoxicity assay with TMZ (white circles) or NEO212 (black circles) using MTT assay in USC02. As shown, NEO212 began to exert cytotoxic activity at concentrations below 50 µM (p<0.05), whereas TMZ was not toxic at 200 µM. Data are expressed as percent cell survival relative to untreated control. All conditions were performed in triplicate. (B) The amount of intracellular TMZ or NEO212 at 1 and 2 hours after treatment of USC02 cells with 100 µM TMZ or NEO212, as measured using MS. Shown is the relative amount of each compound (target) as a ratio to internal standard (IS). Difference between TMZ and NEO212 was significant (p<0.001).
